# Extracts from the Minutes of Atlanta Medical Society

**Published:** 1866-06

**Authors:** 


### *Extracts from the Minutes of Atlanta Medical Society.*

ATLANTA, April 24, 1866.

Reports of Cases being called for—

DR. CONNALLY.—A case of doubtful diagnosis; supposed to be menengitis—perhaps cerebro-spinal menengitis. Survived only about 50 hours after the attack. When first seen, presented no alarming symptom. Had suffered very much from pain in head, but at the time of visit was distressed with nausea and vomiting. Had two copious evacuations during the day; tongue very slightly red; no tenderness of epigastrum. In a few hours drowsiness and slight delirium were manifested, and in this condition died suddenly.

DR. O'KEEFE.—Pulmonary disease; of which he gave the following history and opinions connected with this subject:

April 14th, 1866. Mrs. D., aged 50, a widow lady for 28 years, the mother of one child, and daughter of a Methodist minister; married at the age of 21, and in one year thereafter was delivered of her first and only child. Had good health until three years after her marriage, when she suffered from her first attack of hemoptysis, (a profuse one,) now 26 years ago, of which she has had a repetition as often perhaps as a dozen times—has had a cough more or less constantly since that time. Her menstrual functions were regularly performed until the 43d year of her age, then became somewhat deranged for three years, and finally ceased. During the latter three years the hemoptysis was sometimes vicarious of the menstrual discharge, and the pulmonary symptoms have been more persistent and troublesome since the cessation of the catamenia. There is no hereditary tendency to tuberculous disease in any branch of her family, so far as she is informed, (and she is an intelligent lady) except that her mother had pulmonary symptoms for nine years before her death.

*Present Condition.*—General condition feeble—not much emaciation, though she does not weigh quite as much as she has at former periods of her life; pulse feeble and somewhat accelerated; appetite good; cough frequent, paroxysmal and troublesome; respiration short and quick; expectoration very abundant at times, opaque and thick. At times there is an aggravation of all the pulmonary symptoms, cough, dyspnœa, etc., attended with a febrile condition, which confines her to bed.

*Physical Signs.*—Inspection of the thorax reveals no alteration of its normal contour; both sides are perfectly symmetrical. In all parts of the left lung, the percussion sound, vocal resonance and respiratory murmur are normal, except that the latter is puerile, indicating simply an increased function of the organ—that is to say, the left lung is perfectly healthy. In the right lung there is a *shade* of dullness, more marked over the middle lobe anteriorly, than elsewhere, and this is all the evidence of disease that percussion affords. Auscultation reveals slightly increased vocal resonance, prolonged expiration and imperfectly developed sonorous and sibilant rales under the clavicle; over the balance of the lung anteriorly, laterally and posteriorly, are heard sonorous, sibilant and mucus rales, (the last at the base of the lung posteriorly) variously blended and in various proportions, with bronchial respiration anteriorly. Nothing abnormal in the cardiac region. There was a moderate degree of folicular disease of the posterior wall of the pharynx.

DIAGNOSIS: What shall this be? What shall we call it? Was it or was it not a case of tubercular phthisis? Taking the history and rational symptoms of the case, the conclusion would almost inevitably be reached that it was phthisis pulmonalis ; thus, hemorrhage from the lungs frequently for 26 years ; cough more or less constantly for the same period ; expectoration of muco-purulent matter abundant ; general debility ; pulse feeble and somewhat accellerated, and to cap the climax, her mother had died of pulmonary disease of nine years standing, which, in all probability, was consumption. Now did we not go beyond the history, constitutional and rational symptoms, the case would at once be set down as phthisis. Take the single item of hemoptysis so often repeated for so long a time, and according to our best authorities the diagnosis would be "tuberculosis." I have heard a distinguished authority on this subject, (Prof. Alonzo Clark, of New York city,) announce *ex cathedra*, that he had never found this symptom unassociated with tuberculous deposit in the lungs. Another authority equally distinguished in this department of practical medicine, Prof. Swett, in his work on diseases of the chest, says: "In certain forms of heart disease, in gangren of the lungs, in cancer, in cirrhosis of the lungs, and perhaps occasionally in women, as a form of vicarious menstruation, you may find hemoptysis. But these cases, in addition to their peculiar attendant symptoms, are so rarely met, while hemoptysis from tuberculous disease is so common, that its occurrence will naturally bring with it the presumption that, when hemoptysis exists, tubercles in the lungs exist." Again says the same author : " Cases of simple pulmonary congestion followed by hemorrhage, and by a return of the lung to a healthy condition, have never occured to me." If we consult any other authority on this point, Northern or European, their testimony is singularly unanimous in attaching a formidable importance to this symptom, in its relation to the existence of pulmonary tubercles.

I have prominently singled out this symptom, Mr. President, for the purpose of expressing my humble opinion, that in this climate, so far as my limited observation extends, we are not authorized to attach to it the same fearful import as the authorities just quoted. While it is quite true that hemoptysis is often, alas ! too often, the precursor of the early inevitable doom of the subject of it, still it is equally true, according to my observation, that the history of cases is, not unfrequently, presented to us in which this symptom has repeatedly occurred in early life in connection with other pulmonary symptoms, all of which have disappeared, and the parties have been restored to a good degree of health and lived to middle or advanced life, finally dying of some disease having no connection with the lungs. Now what does the history of such cases teach us? Shall we infer that the hemorrhage depends upon simple congestion of the lungs unconnected with the deposit of tubercles, or shall we conclude that it owes its existence to a tuberculous deposit which has been absorbed, restoring the integrity of the lung, or become dormant and innocuous by its transformation into cretacious or carbonacious matter? The weight of authority is largely in favor of the latter opinion, and having no pathological facts to oppose to it, I adopt it as the most reasonable explanation of the problem. The existence of these chalky concretions, sometimes associated with cicatrices, and more frequently not, at the summit of the lungs, is much more frequent than was formerly supposed, and such cases are, in my opinion, justly regarded as examples of cured consumption. Some interesting facts have been developed in the investigation of this question. In one hundred women who died above sixty years of age of various diseases, in the Salpetriere Hospital of Paris, "fifty one presented the curative indications of tuberculous disease, and chiefly by the formation of chalky concretions." Prof. J. Hughes Burnett has investigated this point by a number of autopsies of persons dying of different diseases, with the result that about one half presented evidences of having had the tuberculous deposit at some period of their lives. That unusually cautious and reliable observer, Prof. Swett, says: "I have known a number of patients during the last fifteen years, who have had the evidences of phthisis, and sometimes in an advanced stage, who finally recovered, and are now in the enjoyment of good health. * * * * * What light does post mortem examination throw upon this subject? For the past fifteen years, I have been in the habit of examining the lungs of all my patients dying of every form of disease, independent of phthisis, for the traces of phthisis that has been cured. I have been astonished at the number of cases which have presented evidences of this favorable result. I can say that it is not uncommon to find in patients who have died of various diseases, and in which no suspicion of tuberculous disease existed at the time of death, cretaceous masses, few in number, at the summit of the lungs, and sometimes, but more rarely, cicatrices in the same situation, and commonly existing with these cretaceous masses. * * * * * * * * This result did not surprise me, as it would have done many of the profession, who believe that tubercles are equivalent to a death warrant. Indeed, I am inclined to think that as the diagnosis of tubercles has become more certain and satisfactory, many cases will be found presenting undoubted evidences of phthisis, which yet recover, and that the common expression, 'The patient could not have had phthisis, because he recovered,' will cease to be believed."

Supposing, then, the case to be phthisis and that these hemorrhages depended upon the tuberculous deposit, we have a case of chronic phthisis. It would then be pertinent to inquire, can consumption last twenty six years? Can this usually formidable disease exist so long without the destruction of the patient? We are justified, in my opinion, by our best authorities, in answering this question in the affirmative. Swett witnessed a case of phthisis which lasted thirty-five years, which was marked by occasional returns of, sometimes, very copious hemorrhage during this long period, and examples could be multiplied indefinitely on this point. The pathological process in a case of chronic or *intermittent* tuberculosis would run about in this wise. A crop of tuberculous exudation takes place in the lungs, congestion is excited thereby which nature attempts to relieve by the rupture of some of the capillary vessels and hence the hemorrhage. We would therefore deduce the practical rule that the influence of the hemoptysis, if moderate, is salutary, and that we need not trouble ourselves to devise remedies for its suppression. Sometimes, however, the hemorrhage is profuse and may endanger life. I have seen one case in which it was the immediate cause of death by asphyxia. Under such circumstances, the blood comes from a vessel of considerable size, which has been destroyed by the tuberculous ulceration which belongs to the third stage of phthisis. This result, however, that is fatal hemoptysis, is extremely rare in my opinion; and another part worthy of consideration is, as a rule, that a consumptive patient with hemoptysis will live longer than one without it; that the process of tuberculization, with its destructive results, is more rapid in that class who have no hemorrhages. It was formerly supposed that hemorrhages from the lungs owed their origin to two conditions, viz: exhalation from a free surface, as the bronchial mucus membrane and rupture of a blood-vessel; microscopical observations, however, have demonstrated that there can be no such thing as an exhalation of blood—that it must always escape from a ruptured blood-vessel. "Its mechanism is simply this: The tuberculous deposit, by pressing upon the capillary vessels of the lungs, obstructs some of these, while others become congested in consequence. These congested vessels, when seated in a mucus tissue, become ruptured with distention, and discharge blood."

But to return to our case. Is it chronic phthisis? The physical signs scarcely establish this opinion. Let us recur to them: Slightly increased vocal resonance, prolonged expiration, sonorous, sibilant and mucus rales, bronchial respiration, with only a *shade* of dullness in a case of 26 years duration, and these signs existed at the middle and base of the lung, as well as its summit. It is well known that a change of the normal percussion sound is one of the most reliable evidences of an established case of phthisis—indeed that a deposit of any extent cannot exist—unless it be of the diffused miliary tuberculous exudation in the interior of the lung, without producing more or less dullness on percussion. There was but a shade of dullness, scarcely worth mentioning, except for the purpose of accuracy—and it seems to me that if the physical evidences of disease which existed in the lung from summit to base were due to a deposit of tuberculous matter—this condition having existed to a greater or less extent for so long a time—we certainly should have a well marked change in the percussion sound.

I am compelled to believe, therefore, after a careful scrutiny of the case in all its bearings, that its main pathological element is chronic bronchitis with dilatation of the bronchial tubes. This view of the case affords, to my mind, a more satisfactory explanation of all the phenomena than any other. Even hemorrhage has been reported as connected with this condition.

In couclusion, Mr. President, I believe that phthisis, although a fatal disease in a large propórtion of cases, is yet curable in some; and that the developments of recent pathological investigation tend to show that it has been cured much more frequently than was formerly thought possible. If the chalky concretions and cicatrices found in the lungs of persons who have died of various diseases be regarded as evidences of cured tubercles, then it would seem that about one half of all that die in advanced life have had the tuberculous deposit at some period of their lives. I believe that as the diagnosis of tubercle becomes more certain and satisfactory, enabling us to recognize the disease in its incipient stage, and justifying us in putting the patient on his guard in regard to his condition, the proportion of cures will be increased, and as has been already said, consumption will cease to be regarded as a death warrant. Just here I would advert to a usage which, to my mind, is full of evil. A man suffers an attack of hemoptysis, his friends and very often, his medical attendant, endeavor to convince him (which is by no means a difficult matter,) that the blood is from his throat, gums, etc., etc., that it is a mere accidental thing and means nothing. This, in my opinion, is all wrong. Hemoptysis has, usually, a fearful import, and the unfortunate victim should be warned of his danger; his habits, occupation and general mode of life should conform to his condition; he should be informed of the serious character of the disease impending over him, which may be averted by proper action on his part. While acting in this fair and candid manner, I should at the same time, assure him that his condition was by no means a hopeless one, indeed, that there was much of hope in it if a timely and proper course be taken by himself. Another evil of equal magnitude is the injudicious use of drugs in these cases. The whole class of nostrums and patent medicines is gone over; every old woman has a remedy that cured just such a case, and alas, even the medical man is sometimes so illy informed as to the true nature and character of his patient's malady, that his digestive functions are almost totally destroyed by mercurials, opiates, nauseants and expectorants.

Rather than submit to this abominable system of drugging, I

would turn the consumptive loose and advise him to lead the life of the savage; to take the rain and wind and frost and snow, and all manner of exposure in the open air. Several years ago I met an intelligent gentleman in Greene county, in this State, who was the traveling agent of several Northern periodicals. He gave me the following history of his health, which I have every reason to believe was strictly correct: He had been a merchant in New York city and lost his health by pulmonary disease, which his physicians pronounced phthisis. The disease progressed until he became bed-ridden, and a tuberculous cavity had formed in the apex of one of his lungs. At this stage of his case, he had hectic, night sweats, and was so feeble that he could scarcely get out of bed. He was informed by his friends and his medical attendants, that his living was a mere question of time. He determined to make a powerful effort for his life, and, in his quaint language, "to die game if possible." He procured a horse, and secured the agency for a few periodicals, merely to afford him an object for traveling, got out of bed, and left the city on horse-back in the fall, with his face Southwards. He rode but a very short distance the first day, a little more the next, and so on, increasing the distance from day to day. On he rode through New Jersey, Pennsylvania, Maryland, Virginia, North Carolina, South Carolina, Georgia and Florida: and as he progressed, he gained in strength and distance travelled every day, until his arrival in Florida, he felt like a new man.

In all this journey, he made a point never to stop at hotels, or taverns, or any other pretentious establishments, but selected, when practicable, the plain, rude farm house—very often the log cabin—where he was sure to get pure air, a clean bed, and simple fare. He groomed his own horse, blacked his own boots, and performed all kinds of drudgery that came in his way, and seldom stopped, even in inclement weather. He spent the winter traveling in the South, mostly in Florida, and returned to New York the following summer, his health being almost restored. His cough had entirely left him, his strength and flesh had returned, and he was in all respects a cured man. His physicians examined his chest, and found that the evidences of the tuberculous cavity had disappeared, and that a cicatrix had taken its place. Eight years had elapsed from the time of the commencement of this history, and its recital to me, all of which he spent in the manner described, spending the summers North and the winters South, but always on horseback in the open air, and in the country. At the time I saw him he seemed to be in excellent health and buoyant spirits, and was altogether, a well-informed companionable gentleman. For several years after this interview, he made me an annual visit on his journey southward, and was still enjoying good health.

Several years ago, it was alleged that the high latitudes of the North-west—Minnesota for example—with their dry atmosphere, and uniformly cold temperature, were more favorable as winter or summer residences for consumptives than the Southern States. My friend tried Minnesota, but could not live there.

Dr. Graves, of Dublin, I think it is, who reported a case of this kind in the journals a few years ago. I quote it from memory, but think the facts are substantially correct. A gentleman from the country brought his son, a young man 20 to 25 years of age, to Dublin, to be examined by Dr. G. The result of this examination proved that he had a tuberculous deposit in a softening condition in the apex of one of his lungs ; i. e. that he was in the second stage of phthisis. The diagnosis was frankly communicated to the father, but not to the patient, (as was then thought,) with its probable consequence in due course of time. Dr. Graves saw nor thought no more of the case for about one year, when a young man of vigorous and robust health called on him. He remarked, "You do not recognize me, Doctor?" "No, sir." "I am the young man brought to you by my father, (giving his name,) about a year ago; you see I am well." The Dr. could scarcely believe his senses, but becoming satisfied of his identity, examined his lungs and found that all traces of disease had disappeared. He asked him what he had done, how he had lived, and how he got cured. His patient replied that he had overheard the diagnosis as communicated to his father, and having, as he supposed, but a short time to live, he would live it fast and make the most of it. He hunted and fished in the bogs and swamps of Ireland every day through the winter, frequently in water waist high, from morning to night; he paid no attention to weather, and lived literally a fast life. To an inquiry of the Dr. if he had drank any, he replied that he had scarcely went to bed sober. Should our consumptive patients commence, at the very incipiency of the disease, some such mode of life as that sketched in these two cases, I verily believe that its mortality would be greatly diminished, and our profession relieved from the stigma which now attaches to it.

Dr. Word presented a specimen of *tape worm*, (*Tœnia Solium*,) 8 feet long. The patient from whom he obtained it, was a child 5 or 6 years old, and had been afflicted for 3 years past with this parasite, occasionally discharging fragments of the worm. The case had been under Dr. Word's charge since August last, during which time frequent efforts had been made to dislodge the worm. *Kosso*, *male fern*, *Turpentine* and other approved remedies had been tried without avail. Better success had attended the use of common *Alum* in this case than any other remedy; from this remedy portions of the worm had been discharged at different times. 20 grs. of alum in solution, given 3 times a day, never failed to relieve the distressing symptoms of the case, yet it was only when the *alum* was given in conjunction with melted lard that any portion of the worm was discharged. 20 grs. of the alum with an ounce of lard, acted powerfully as a purgative, and seldom failed to bring away portions of the worm. Dr. Word expressed the opinion that *alum* was a much more valuable article of the materia medica than is commonly supposed. He had observed that it possessed the power of controlling the heart's action in a marked degree. A solution of 1 ʒ in a glass of water, and given in doses of a tablespoonful every half hour, controled the most active palpitations of the heart, whether resulting from functional or organic cause. He had not yet tested satisfactorily in practice, his views upon this point, but had strong hopes, that though comparatively a harmless and innocent article, it would prove to be an *arterial sedative* of as great power as *veratrum viride*.

